# Gauging the Threat: The First Population Estimate for White Sharks in South Africa Using Photo Identification and Automated Software

**DOI:** 10.1371/journal.pone.0066035

**Published:** 2013-06-12

**Authors:** Alison V. Towner, Michelle A. Wcisel, Ryan R. Reisinger, David Edwards, Oliver J. D. Jewell

**Affiliations:** 1 Dyer Island Conservation Trust, Great White House, Kleinbaai, Gansbaai, South Africa; 2 Department of Zoology, University of Cape Town, Rondebosch, South Africa; 3 Mammal Research Institute, Department of Zoology and Entomology, University of Pretoria, Hatfield, South Africa; Università degli Studi di Napoli Federico II, Italy

## Abstract

South Africa is reputed to host the world’s largest remaining population of white sharks, yet no studies have accurately determined a population estimate based on mark-recapture of live individuals. We used dorsal fin photographs (fin IDs) to identify white sharks in Gansbaai, South Africa, from January 2007 – December 2011. We used the computer programme DARWIN to catalogue and match fin IDs of individuals; this is the first study to successfully use the software for white shark identification. The programme performed well despite a number of individual fins showing drastic changes in dorsal fin shape over time. Of 1682 fin IDs used, 532 unique individuals were identified. We estimated population size using the open-population POPAN parameterisation in Program MARK, which estimated the superpopulation size at 908 (95% confidence interval 808–1008). This estimated population size is considerably larger than those described at other aggregation areas of the species and is comparable to a previous South African population estimate conducted 16 years prior. Our assessment suggests the species has not made a marked recovery since being nationally protected in 1991. As such, additional international protection may prove vital for the long-term conservation of this threatened species.

## Introduction

White sharks *Carcharodon carcharias* are widely distributed apex predators which are known to undertake extensive oceanic migrations [1]–[6]. They also exhibit semi-annual site fidelity to predictable coastal locations often associated with pinniped colonies, effectively pooling individuals to locations, or aggregation areas, accessible to researchers [7]–[9]. These aggregation areas have provided a unique opportunity to estimate white shark populations utilising mark-recapture methods [9], [10].

Photo Identification (photo ID) has been developed as a non-invasive method of mark and recapture in which distinctive features of an individual can be used to recognise it against the rest of the population during different samples, over extensive time periods. This method is particularly appropriate when examining vulnerable species or populations, from invertebrates [11], [12] to tigers [13], marine mammals [14]–[16] and sharks [17]–[19]. The first dorsal fin of white sharks is often characterised by distinctive shapes, notches, scaring and pigmentation patterns, which can be used to recognise individuals over many years [7], [19]. From such photo ID data, mark recapture methods can be applied to estimate population sizes, given that the model’s basic assumptions are met adequately [14], [20].

Previous white shark population estimates suggest that white shark numbers are small relative to other apex predators [20]. Nasby-Lucas & Domeier [9] identified a minimum of 142 individual white sharks using photographic identification at Guadalupe Island (GI) from 2001–2009. Chapple *et al.* [10] used mark recapture with a closed population model and estimated the regional population of white sharks in California to be 219 animals. However Sosa-Nishizaki *et al.* [20] and Nasby-Lucas & Domeier [9] contest this estimate on the basis that the use of a closed model was inappropriate, there was no account for inshore recruitment [21], [22], a lack of sampling from Año Nuevo Island (a major aggregation site for Californian white sharks) [23] and an insufficient sampling period for the methods to reflect appropriate trends. Cliff *et al.* [24] used mark and recapture of white sharks tagged with spaghetti tags that were captured and killed in the KwaZulu-Natal (KZN) Sharks Board nets to estimate a population of 1279 (CV 24%) between Struisbaai, Western Cape and Richards Bay, KZN. The weakness of this method, however, was that the sharks used for the estimate were killed and were then no longer part of the population.

Few studies have attempted to estimate the South African population using mark and recapture techniques of living individuals [25]. The study site of Gansbaai was selected to make a first estimate of the current population of white sharks in the region. Gansbaai is a world-recognised white shark aggregation site and is the only location in the world where cage diving trips operate daily, weather permitting [26]. Thus, this aggregation site provided an ideal area to collect dorsal fin images of a range of age classes and both sexes without seasonal paucities. Additionally, several individual white sharks from this region have been identified at other aggregation sites, with connectivity documented between False Bay, Mossel Bay, KwaZulu Natal, and Western Australia [1], [19], [27]. This suggests that Gansbaai is a major aggregation area for white sharks in the Southern African region allowing for an accurate population estimate from a single site.

## Methods

We encountered white sharks at two aggregation areas in Gansbaai (Geyser Rock and Joubertsdam). Trips were permitted by the Department of Environmental Affairs, Oceans & Coasts (formerly operating under Marine and Coastal Management); data collection was un-invasive and required no further ethical clearance. We attracted sharks to one of two cage diving vessels run by Marine Dynamics shark tours with a fish bait, seal decoy, and a scent trail created by a mixture of fish products and sea water. Tours were weather dependent and biased towards areas of high white shark abundance. We obtained images of white shark dorsal fins during 1647 trips coinciding with ca. 4120 hours of sampling effort (∼2.5 hours average) from January 2007 – December 2011.

We imported dorsal fin images into Picasa, a photograph editing programme (picasa.google.com). Images were organised by date, cropped and assigned a 1–6 quality ranking defined in Gowans & Whitehead [28]. A ranking of 6 is considered to be a fin ID of the highest quality, i.e. the fin is entirely clear of the water, square to the camera, with good focus, lighting and adequate zoom. Q5 is considered high quality with only one of the previous requirements in Q6 lacking. The Q ranking decreases as the quality of photo ID decreases. We found that only photographs Q4 and higher provided sufficient information to recognise individuals and allow “recapture” between sightings. Such photographs (n  = 1683) were then imported into DARWIN dorsal fin ID software [29]. Each dorsal fin was traced and assigned a fixed spacing of points along the leading and trailing edges. This trace was compared to the entire catalogue and ranked by probability of a match. Final matching was confirmed by eye as pigmentation, scarring, freckles or fin changes over time can be accounted for. Fins that could not be matched to a fin already existing in the catalogue were assigned a unique ID code corresponding with the first date of sighting and fin order of that trip (e.g., 20110601-2 for the second shark on 1 June 2011) and then added. Inputted fins that matched a catalogue fin were assigned the ID code of that matched individual and also added. Data from DARWIN was exported to MS Excel where individual abundance and occasionality could be assessed.

We performed mark-recapture analyses of the sighting histories of recognisable individuals using Program MARK [30], which uses Maximum Likelihood models to estimate population parameters [31]. We pooled photographic sightings into quarterly sampling intervals after comparing the results for various sampling intervals. Population closure was not a reasonable assumption and therefore we used the open-population POPAN parameterisation [32], [33] to estimate population parameters. In this parameterisation, *N* represents the size of a superpopulation; which can be thought of as either the total number of individuals available for observation at any time during the study or as the total number of animals ever in the sampled area between the first and last occasion of the study [34]. The parameter *Φ* denotes apparent survival rate, *p* is the probability of observation and *b* represents the probability that an animal from the superpopulation enters the sub-population (sub-population referring to the animals occurring in the study area). In model notation, the subscripts *t* and**.** represent time-dependent and constant parameters, respectively, [35] and the initial analysis is based on the fully time-dependent/Cormack-Jolly-Seber (CJS) model {*Φ_t_ p_t_ b_t_*}. The first step in the analysis involves Goodness-of-Fit (GOF) tests for the CJS model using Program RELEASE [36] to validate model assumptions. Models were constructed for combinations of time-dependence and consistency for each parameter and the most appropriate model was selected using the small sample corrected Akaike Information Criterion (AIC_c_) [37]. Based on the GOF results of TEST 2+ TEST 3 in RELEASE a post-hoc variance inflation factor (*ĉ*) may be estimated to adjust for extra-binomial variation in the data, resulting in a quasi-Akaike Information Criterion (QAIC_c_).

## Results

**Figure 1:**
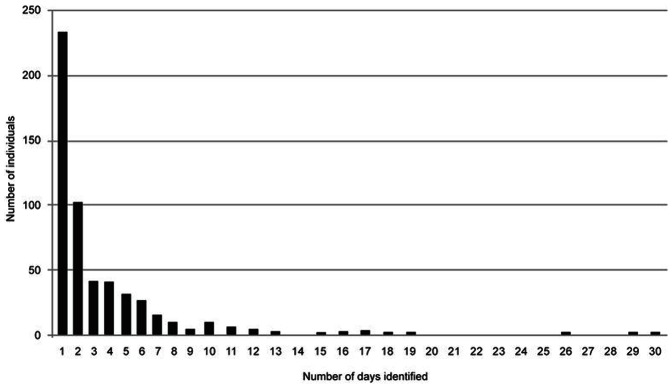
Sighting frequency distribution of photographically identified white sharks in the Gansbaai region (2007–2011).

**Figure 2:**
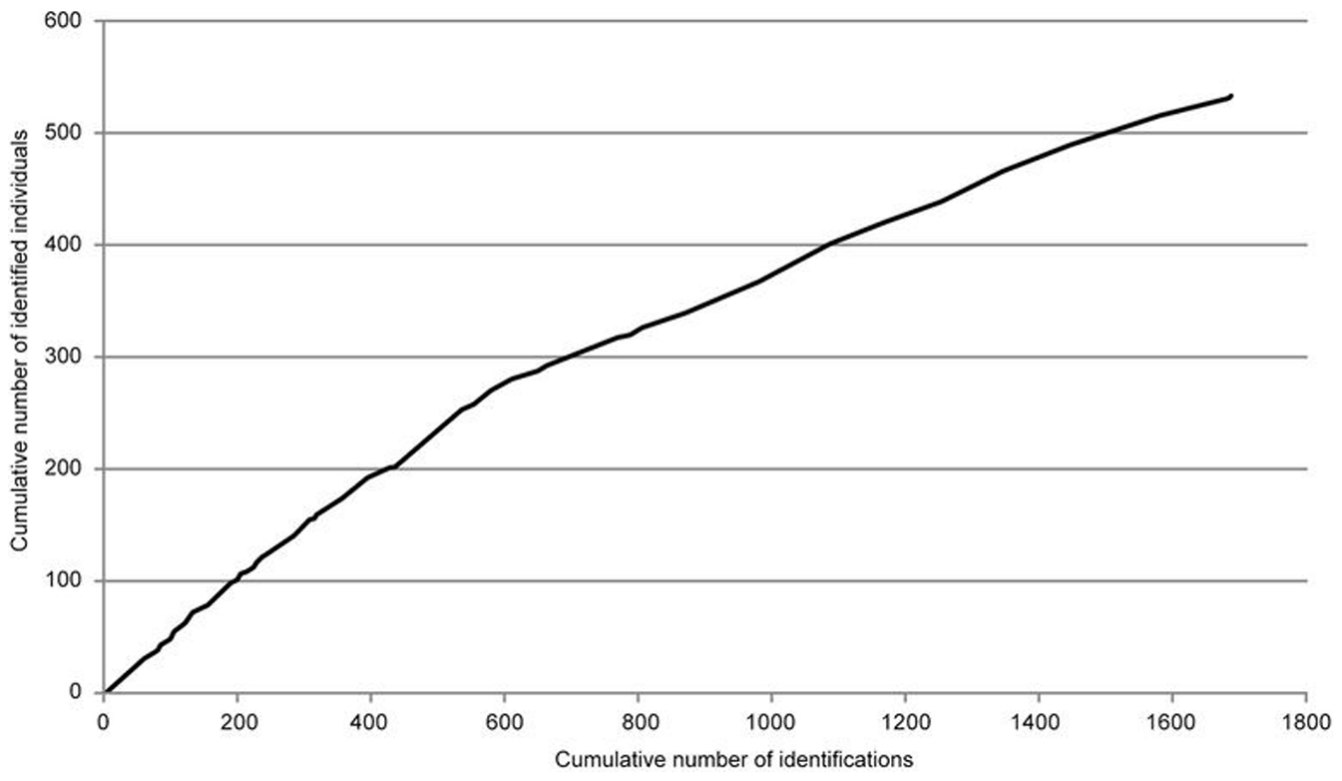
Discovery curve for photographically identified white sharks in the Gansbaai region (2007–2011).

We identified 532 unique individuals which were included in the population size analyses. Figure 1 shows the sighting frequency distribution of these animals and figure 2 shows the discovery curve – or cumulative number of identified individuals – as the study progressed. Of the eight models tested, two did not converge. Both of these contained *b_._* (constant probability of entry); the two other models containing *b_._* did converge, but had very large quasi-deviances, indicating that constant probability of entry was not a reasonable assumption. For the remaining four models, model choice criteria as well as abundance estimates and parameter estimates are shown in Table 1. Based on the result of TEST 2+ TEST 3 in Program RELEASE (Table 2), a variance inflation factor of *ĉ*  = 1.36 was estimated and applied, indicating only slight over dispersion in the data [31]. According to the QAIC_c_ scores, model {**ϕ**_._ *p*_t_ *b*_t_} (constant survival, time-varying probability of capture and probability of entry) was the most parsimonious. This model estimated the superpopulation size at 908 individuals (95% confidence interval  = 808–1008). No models had a ΔQAIC_c_ <2 units, which would have indicated that they were also likely [37]. Some violation of underlying open-population mark-recapture assumptions was evident (Table 2). Significance of TEST 3 (*p*  = 0.006) and one of its components (3.SR; *p*  = 0.0001) indicate unequal survival probabilities among photographically captured animals.

**Table 1:** Model choice criteria and abundance estimates (*N*) for four models tested in a mark-recapture analysis of individual sighting histories of white sharks in the Gansbaai region (2007–2011), using the open-population POPAN parameterisation in Program MARK.

Model	QAIC_c_	ΔQAIC_c_	Model Likelihood	Parameters	Quasi Deviance	N	95% Confidence Interval		CV
							Lower	Upper	
**φ**_._ P_t_ b_t_	1 719.88	–	1	35	0	908	808	1 008	0.056
**φ**_t_ p_t_ b_t_	1 746.38	26.50	0	53	0	823	717	929	0.066
**φ**_._ p_._ b_t_	1 791.19	71.31	0	14	0	950	853	1 048	0.052
**φ**_t_ p_._ b_t_	1 802.91	83.03	0	33	0	956	851	1 062	0.056

See text for criteria.

**Table 2:** Program RELEASE goodness-of-fit results for the fully time-dependent/Cormack-Jolly-Seber model tested in a mark-recapture analysis of individual sighting histories of white sharks in the Gansbaai region (2007–2011), using the open-population POPAN parameterisation in Program MARK.

Test	X^2^	Df	P	Ĉ
2	51.86	47	0.2900	–
3	56.75	33	**0.0062**	–
3.SR	48.68	18	**0.0001**	–
3.SM	8.07	15	0.9209	–
2+3	108.61	80	**0.0184**	1.36

Shaded *p*-values are significant at α  = 0.05.

## Discussion

**Figure 3:**
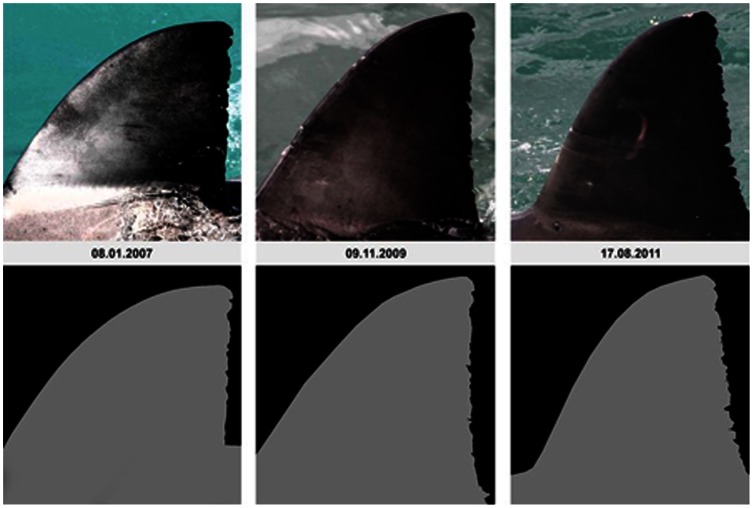
Study animal ‘Darwin’, photographed numerous times from 2007–2011. This fin clearly demonstrates how small additions/changes to notches occurring on the back of the fin can distinguish it from early photos, increasing the probability of later fin IDs being identified as multiple sharks.

**Figure 4:**
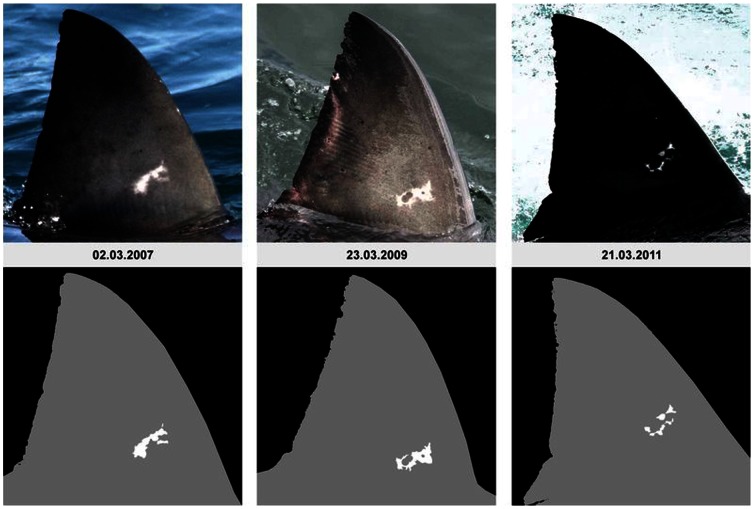
Nine photographs were obtained for the study shark ‘Zebra’ between 30/08/2007 and 28/06/2011. During this time, the fin sustained significant damage to both its front and back areas, altering its profile. This affects the number of notches down the back of the fin.

**Figure 5:**
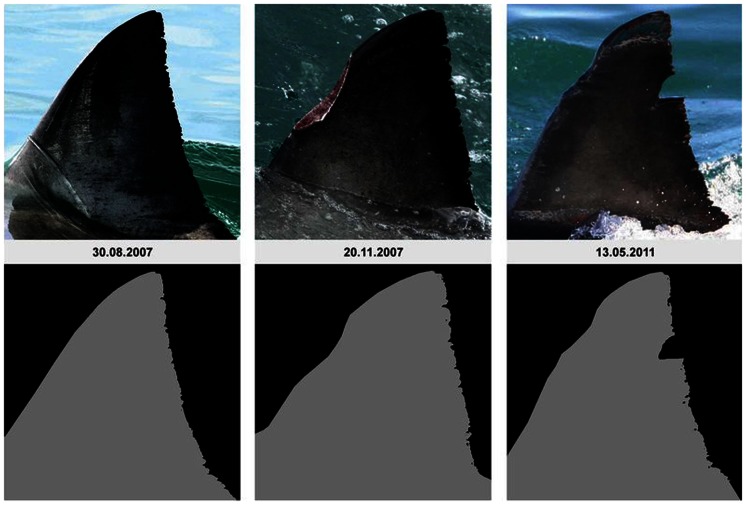
Study shark ‘Vindication’, one of the few sharks to be photographed at least once in every year from 2007–2011. Vindication features numerous, subtle changes in harsh contrast to the minimal, significant scars on the other examples above. On Vindication’s dorsal fin, white pigmentation ‘roise’ changes occurred slowley over time.

Unlike Chapple *et al.* [10], we found the computer program DARWIN suitable for matching and cataloguing white shark dorsal fins within a large dataset. While DARWIN had infrequent considerable errors in ranking fins, we considered this flaw minor when compared to the human error of matching by eye alone. Confirming this, we found that many dorsal fin trailing edges changed dramatically from one sighting to another, an aspect we were only able to detect as DARWIN accounts for more than just the notches in the trailing edge of the fin. In most cases, the leading edge and top quarter of the fin exhibited very little change (Fig. 3), whereas the bottom ¾ of the fin can be unrecognisable between sightings (Fig. S1). We also observed changes to entire fin shapes (Fig. 4). We were able to track these changes over time due to consistent sampling effort, allowing us to consider fresh damage or changes to fins when cataloguing. Without consistent effort, it is possible that individual fins which changed would not be successfully matched to the same individual shark, thus fin IDs from the same shark over time can be counted as multiple individuals resulting in an over-estimation of animals. In addition, pigmentation patterns, or ‘rosies’ [26], [38], were found to change over time in most cases (Fig. 5) which is similar to the pigmentation changes that have been described to occur around the lower caudal areas of white sharks in south Australia [38]. The amount of fin degradation in the lower quarters of the fin or changes in pigmentation patterns do not seem to relate to the size/class of the individual upon first sighting. However, presence of copepod parasites along the trailing edge of the fin did seem to lead to initial notch formation. These changes in fin morphology highlight that previously proposed methods based on counting fin notches (i.e. ‘The Rutzen Method’ in O’Connel *et al.* [39]; Andriotti *et. al.* [40]) are unreliable for long-term mark-recapture analysis.

A common bias in many mark-recapture studies is capture heterogeneity [41]. In our study we attracted sharks by bait, thus some individuals may have become ‘trap happy’ or ‘trap shy’ over time [41]. This may lead to bias on estimates, but the effects of baiting on individual sharks remains undetermined [27], [42]. To address this, future work should focus on the effects of shyness or boldness in individual white sharks and assess whether they are more or less likely to appear close to a baiting vessel over time as well as incorporating such heterogeneity in behaviour in population size estimation.

Our population estimate for white sharks in Gansbaai is considerably higher than those obtained for other aggregation areas [9], [10], [20], supporting claims that South Africa has the largest remaining population of coastal white sharks [25]. Our estimate is comparable to that given by Cliff *et al.* [24]. This is not surprising, as most white sharks that utilise Gansbaai aggregations also move into KZN shark netted areas [43]. Unfortunately, dorsal fin photos of white sharks killed in KZN shark nets were not collected during this study period, therefore we cannot compare the living fin-ID population of Gansbaai to the culled population in KZN [24], [44]. There are 11 years between the end of Cliff et al.’s [24] data collection and the beginning of sampling in Gansbaai (this study). This suggests white shark numbers have not shown marked recovery from; 1) the deployment of shark nets and drum lines along the KZN coastline in 1952, which are still in place to date [44]; 2) the heavy fishing pressures white sharks experienced in the 1970’s and 80’s [8]; and 3) a lack of protection in neighbouring Mozambique [43]. Despite the species being protected since 1991 [8], such a low estimate and lack of recovery rate suggests the Southern African white shark is not receiving adequate protection for population growth. These results highlight the need for effective protective measures within the entire home range of the Southern African white shark.

## Supporting Information

Figure S1 Study shark ‘Demon’, demonstrating signficant change in the lower three quarters of the trailing edge. Despite having a large injury to the trailing ege of the dorsal fin, the fin identification can still be matched by using the shape of the leading edge and the top quarter of the fin.
(TIF)
