# Correction: Gauging the Threat: The First Population Estimate for White Sharks in South Africa Using Photo Identification and Automated Software

**DOI:** 10.1371/annotation/bb25e7cb-12f7-42e4-9d26-07b9bda87ecc

**Published:** 2013-06-14

**Authors:** Alison V. Towner, Michelle A. Wcisel, Ryan R. Reisinger, David Edwards, Oliver J. D. Jewell

Figures 4 and 5 are reversed; they contain the incorrect figure legends.

- The following is the correct figure 4 legend:

Figure 4. Study shark ‘Vindication’, one of the few sharks to be photographed at least once in every year from 2007–2011. Vindication features numerous, subtle changes in harsh contrast to the minimal, significant scars on the other examples above. On Vindication’s dorsal fin, white pigmentation ‘rosie’ changes occurred slowly over time.

- The following is the correct figure 5 legend:

Figure 5. Nine photographs were obtained for the study shark ‘Zebra’ between 30/08/2007 and 28/06/2011. During this time, the fin sustained significant damage to both its front and back areas, altering its profile. This affects the number of notches down the back of the fin. 

