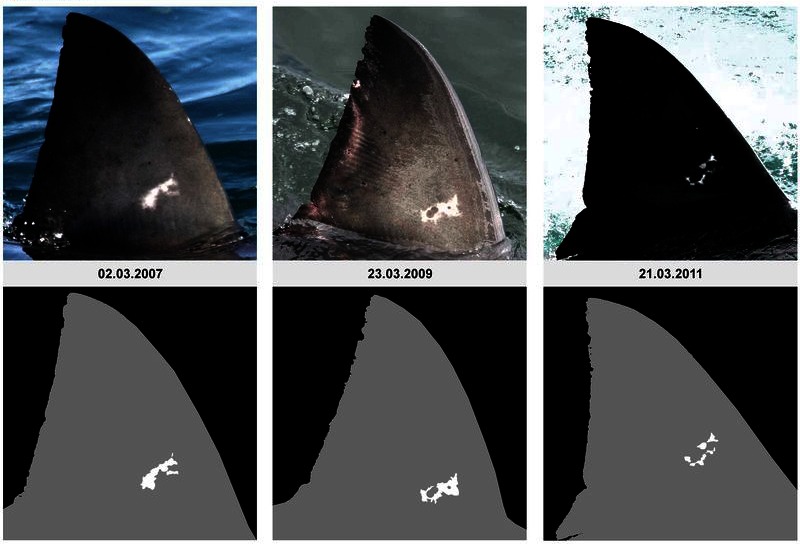# Correction: Gauging the Threat: The First Population Estimate for White Sharks in South Africa Using Photo Identification and Automated Software

**DOI:** 10.1371/annotation/2ac91258-bfcc-40f4-adee-3f855b1a4be7

**Published:** 2013-06-17

**Authors:** Alison V. Towner, Michelle A. Wcisel, Ryan R. Reisinger, David Edwards, Oliver J. D. Jewell

The previous correction notice is inaccurate. The images of Figures 4 and 5 are placed in reverse order. The figure legends to Figures 4 and 5 are correct. See the following links for the correct versions of the figures.

Figure 4: 

**Figure:**
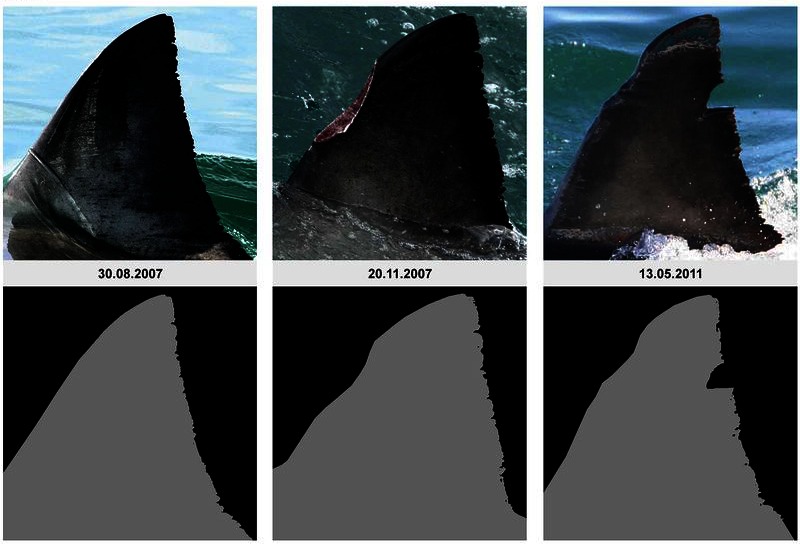


Figure 5: 

**Figure:**