# Supplementary figures and images for: Gauging the Threat: The First Population Estimate for White Sharks in South Africa Using Photo Identification and Automated Software
Source: PLoS One. 2013 Jun 12;8(6):e66035. doi: 10.1371/journal.pone.0066035 (PMC3680411; doi:10.1371/journal.pone.0066035)

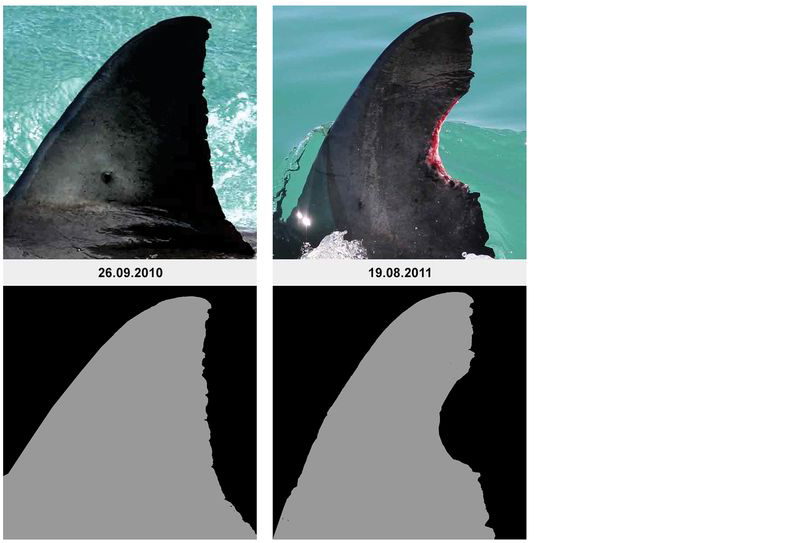

Supplement: Figure S1 — Study shark ‘Demon’, demonstrating signficant change in the lower three quarters of the trailing edge. Despite having a large injury to the trailing ege of the dorsal fin, the fin identification can still be matched by using the shape of the leading edge and the top quarter of the fin. (TIF) [file pone.0066035.s001.tif]
